# The Influence of Religious Affiliation on Participant Responsiveness to the Complete Health Improvement Program (CHIP) Lifestyle Intervention

**DOI:** 10.1007/s10943-015-0141-3

**Published:** 2015-10-15

**Authors:** L. M. Kent, D. P. Morton, E. J. Ward, P. M. Rankin, R. B. Ferret, J. Gobble, H. A. Diehl

**Affiliations:** Lifestyle Research Centre, Avondale College of Higher Education, 582 Freemans Drive, Cooranbong, NSW 2265 Australia; Spirituality and Worship Research Centre, Avondale College of Higher Education, Cooranbong, NSW 2265 Australia; Medical Nutrition Therapy Northwest, Clackamas, OR 97015 USA; Lifestyle Medicine Institute, 25805 Barton Rd, Bldg. A, Ste. 106, Loma Linda, CA 92354 USA

**Keywords:** CHIP, Seventh-day Adventist, Chronic disease, Lifestyle intervention, Diet

## Abstract

Seventh-day Adventist (SDA) and non-SDA (21.3 and 78.7 %, respectively) individuals (*n* = 7172) participating in the Complete Health Improvement Program, a 30-day diet and lifestyle intervention, in North America (241 programs, 2006–2012) were assessed for changes in selected chronic disease risk factors: body mass index (BMI), blood pressure (BP), pulse, lipid profile and fasting plasma glucose (FPG). Reductions were greater among the non-SDA for BMI, pulse and blood lipids. Furthermore, the majority of non-SDA in the highest risk classifications for BP, lipids and FPG, but only some lipids among SDA, were able to show improvement by 20 % or more.

## Introduction

The burden from chronic disease is rising rapidly and represents one of the major health challenges in the USA, with half of all deaths each year attributed to heart disease, stroke, diabetes and cancer (CDC 2012). Chronic diseases also carry a major fiscal burden. The direct (medical) and indirect (productivity) costs of cardiovascular disease alone are projected to increase from $450 billion in 2010 to more than $1 trillion by 2030 (Heidenreich et al. 2011). The development of chronic disease has been strongly linked to lifestyle, particularly inappropriate dietary patterns and physical inactivity (Roberts and Barnard 2005).

Research on the health of Seventh-day Adventists (SDA) since the 1950s has shown that they appear to enjoy low rates of chronic diseases, resulting in lower total mortality, despite living in areas where chronic diseases are prevalent (Willett 1999).

## Who are the SDA?

While Seventh-Day Adventism has now become a worldwide religion with more than 18 million members, this conservative religious group was first organized as a denomination in 1863 in the eastern USA (Fraser 2003; SDA 2014). In that same year, SDA also began to emphasize the role of lifestyle in promoting health, happiness and enhanced spirituality (Fraser 2003). While Adventists do not believe that good health practices are related to religious virtue, they do believe that these choices are a valuable spiritual discipline (Fraser 2003); a tenet found in the Judeo-Christian belief system. In effect, Adventists recognize the indivisible unity of body, mind and spirit, with the health of each component being so integrated and interrelated that what affects one affects the functioning of the whole being. Consequently, SDA health philosophy is built around the holistic biblical notion that the human body is the temple of the Holy Spirit; therefore, it is to be cared for intelligently (Fraser 2003). Along with adequate exercise and rest, Adventists are encouraged to adopt the most healthful lifestyle, which includes a diet that is vegetarian but avoids caffeine-containing beverages, rich and highly refined foods, hot condiments and spices, and abstains from Biblically unclean foods, as well as alcohol, tobacco and narcotics (Fraser 2003). The dietary health laws found in Leviticus are the basis for Adventist health reform, but the principles were expanded by Ellen White, the church’s primary health reformer, commencing in 1863. In addition, as propounded by Bandura (2001), the social support and knowledge derived from belonging to a group with similar values help members of the SDA church adhere to the prescribed health behaviors that are not practiced by mainstream society.

## The Complete Health Improvement Program (CHIP)

The SDA church has placed great emphasis not only on traditional health care, but also on education. It owns and runs more than 300 highly regarded hospitals and nursing homes/retirement centers, as well as many clinics, dispensaries and orphanages around the world, including the USA. Not only does the church promote its lifestyle prescriptions to its members, but it is also active in delivering scientifically based health promotion and lifestyle modification programs to the community. One such program is the Complete Health Improvement Program (CHIP). CHIP, developed in 1986, is in harmony with the prescriptive lifestyle of the SDA church and is based on scientific principles (Morton et al. 2013). It has been delivered by either health professionals or trained volunteers in various workplaces, community and medical settings (Aldana et al. 2008; Diehl 1998; Englert et al. 2007; Morton et al. 2013). It has a strong educative component to change the participants’ attitudes toward healthy living, and health literacy around nutrition and health behaviors has been shown to significantly improve as a result of the program (Aldana et al. 2008). Participants are educated on the etiology of chronic disease and the benefits of positive lifestyle choices, with particular attention given to diet and physical activity. Sessions on overcoming barriers to change, developing emotional intelligence and providing participants with strategies [self-monitoring, goal setting and problem solving (including addressing unsupportive social and physical influences)] for behavior change maintenance, in an environment that provides social support, are also included. The CHIP sessions were structured around a process of learn, experience, reflect. The use of the supplied resources means that the program delivery is consistent in each location.

CHIP has been demonstrated to achieve meaningful reductions in selected risk factors—body mass index (BMI), blood pressure (BP), total cholesterol (TC), low-density lipoprotein (LDL), triglycerides (TG) and fasting plasma glucose (FPG)—for cardiovascular disease and type 2 diabetes mellitus among large cohorts from several countries including the USA (Aldana et al. 2008; Diehl 1998; Englert et al. 2007; Morton et al. 2013).

There have been no studies that have explored whether non-SDA can achieve improved health outcomes comparable to SDA following an intensive lifestyle intervention delivered in a Christian setting, with supportive religious community. Given their beliefs on health and that SDA have lower rates of disease than the general population, it is expected that they would have less at-risk biometrics than non-SDA at the commencement of such an intervention. Further, given that SDA should have lower risk factors for chronic disease, it is expected non-SDA would make greater improvements in their risk factors than SDA. The aim of the present study was to examine whether the faith-based intervention, CHIP, can reduce selected chronic disease risk factors in the wider society similarly to within the SDA church.

## Methods

### Study Participants

The CHIP intervention previously described in detail (Aldana et al. 2008; Diehl 1998; Englert et al. 2007; Morton et al. 2013) was delivered to 7166 participants, who had self-selected to participate in the program between January 2006 and September 2012. A total of 241 CHIP interventions (mean group size 30, range 1–203) were conducted at 163 venues throughout North America over this period. There were no inclusion/exclusion criteria other than the participant being able to pay a $200 program cost. By comparison, weight loss diets typically cost more than this per annum (ABC  2014). Participants were invited to attend the intervention through word of mouth invitation, local media avenues and referrals from healthcare providers. As indicated by baseline characteristics (Tables 1 and 2), the participants comprised a more at-risk and ill demographic compared to the general North American population (CDC 2014a, WCIN 2014). The (name of the ethics committee removed for blinding) Ethics Committee (IRB) approved the study.

**Table 1:** Baseline characteristics of SDA and non-SDA, North America, 2006–2012

Factor	*N*	Non-SDA (mean ± SD)	*N*	SDA (mean ± SD)	Test statistic	*p* value
Male	1881	33.3 %	513	33.7 %	*Χ* ^2^(1) = 0.071	0.790
Age	5643	57.4 ± 12.8	1521	57.5 ± 13.8	*t*(2276) = −0.387	0.699
Married	3493	73.7 %	1121	75.5 %	*Χ* ^2^(3) = 5.896	0.117
Smoker	133	0.6 %	9	2.6 %	*Χ* ^2^(4) = 96.74	<0.001
Weight (lb)	5132	194.7 ± 50.2	1365	186.3 ± 51.5	*t*(6495) = 5.470	<0.001
BMI (kg/m^2^)	5127	31.48 ± 7.42	1363	30.10 ± 7.39	*t*(6488) = 6.103	<0.001
SBP (mm Hg)	5088	133.8 ± 19.1	1364	132.4 ± 19.3	*t*(6450) = 2.292	0.022
DBP (mm Hg)	5084	79.91 ± 11.41	1366	79.28 ± 11.57	*t*(6448) = 1.785	0.074
TC (mg/dL)	5218	193.2 ± 41.9	1400	190.4 ± 41.0	*t*(6616) = 2.264	0.023
LDL (mg/dL)	5090	116.7 ± 35.5	1378	114.5 ± 35.6	*t*(6466) = 2.245	0.041
HDL (mg/dL)	5214	49.20 ± 15.28	1400	48.10 ± 13.55	*t*(2442) = 2.604	0.009
TC:HDL ratio	5212	4.215 ± 1.372	1400	4.224 ± 1.400	*t*(6610) = −0.225	0.822
TG (mg/dL)	5206	142.9 ± 89.0	1399	143.4 ± 89.7	*t*(6603) = −0.165	0.869
FPG (mg/dL)	5181	102.9 ± 30.7	1381	97.78 ± 26.41	*t*(2470) = 6.146	<0.001
Pulse (beats/min)	4970	71.71 ± 11.01	1335	70.65 ± 10.67	*t*(6303) = 3.147	0.002

**Table 2:** Mean changes in selected risk factors for non-SDA and SDA from baseline to 30 days, North America, 2006–2012

Risk factor	Non-SDA		SDA		*p* value^4^
	Mean change^1^ (95 % CI)^2^	% change^3^	Mean change^1^ (95 % CI)^2^	% change^3^	
Weight (lb)	−6.489 (−6.669, −6.309)**	−3.3	−5.862 (−6.185, −5.532)**	−3.1	0.001
BMI (kg/m^2^)	−1.039 (−1.067, −1.010)**	−3.3	−0.933 (−0.984, −0.882)**	−3.1	<0.001
SBP (mm Hg)	−7.158 (−7.539, −6.777)**	−5.3	−7.988 (−8.671, −7.304)**	−6.0	0.038
DBP (mm Hg)	−4.216 (−4.475, −3.957)**	−5.3	−4.144 (−4.607, −3.680)**	−5.2	0.790
TC (mg/dL)	−21.21 (−21.91, −20.51)**	−11.0	−18.50 (−19.76, −17.24)**	−9.7	<0.001
LDL (mg/dL)	−15.07 (−15.68, −14.47)**	−12.9	−13.25 (−14.33, −12.17)**	−11.6	0.004
HDL (mg/dL)	−4.198 (−4.404, −3.993)**	−8.5	−3.449 (−3.818, −3.079)**	−7.2	0.001
TC: HDL ratio	−0.138 (−0.159, −0.116)**	−3.3	−0.132 (−0.171, −0.093)**	−3.1	0.800
TG (mg/dL)	−10.89 (−12.40, −9.373)**	−7.6	−12.76 (−15.47, −10.05)**	−8.9	0.238
FPG (mg/dL)	−6.366 (−6.779, −5.953)**	−6.2	−5.580 (−6.324, −4.837)**	−5.7	0.071
Pulse (beats/min)	−2.558 (−2.816, −2.301)**	−3.6	−3.129 (−3.590, −2.668)**	−4.4	0.035

^1^After adjusting for gender, age, smoking, marital status and baseline biometric

^2^** Mean change *p* value < 0.001

^3^% change = [(mean at 30 days − mean at baseline)/mean at baseline] × 100

^4^Significance of change between non-SDA and SDA

### Facilitator Information

The CHIP programs were conducted by volunteer facilitators, sourced primarily through the Seventh-day Adventist Church, who had an interest in positively influencing the health of their local community. All volunteers were required to undergo 2 days of training to learn about the CHIP intervention and develop group facilitation skills. There were no educational requirements or selection criteria for the volunteer facilitators. The educational component of the CHIP intervention was presented through the pre-recorded videos. The role of the volunteer facilitator was to organize the meetings and facilitate discussion.

### Description of CHIP

The CHIP intervention involved 16 group sessions conducted in a community setting over 30 days (Diehl 1998; Englert et al. 2007). The program encourages and supports participants to move toward a low-fat (<15 % of calories from fat), ad libitum plant-based diet over 30 days, with emphasis on the daily whole-food consumption of grains, legumes, fruits and vegetables and water (2–2.5 L), while limiting intake of added sugar, sodium and cholesterol (40 g, 2000 and 50 mg, respectively). This eating style is high in nutrient density and fiber yet low in energy density. In addition, the program advocates that participants engage in 30 min of moderate physical activity daily (walking) and practice stress management techniques (life balance, sleep, rest).

Each of the 16 group sessions, delivered 4 days per week for 30 days, was approximately 1 h in duration, with approximately half of the session involving the viewing of a pre-recorded educational video and the other half constituting group activities, such as cooking demonstrations, physical exercises and discussion. Participants were deemed to have completed the intervention if they attended a minimum of 13 of the 16 sessions and completed the baseline and 30-day assessments. This number of sessions was set so as to equate to more than 80 % of the program. Following completion, participants were invited to attend ongoing monthly follow-up sessions to reinforce lifestyle behavior changes and build a network of support and ongoing education. For a discussion on the development and detailed description of the CHIP program, the reader is referred to the review article by Morton et al. (2014).

### Data Collection and Reporting

Before participating in the CHIP intervention (baseline) and again at its conclusion (post-intervention), the participants’ height, weight, SBP and DBP were taken and fasting (12-h) blood samples were collected by registered health professionals. The same scales and sphygmomanometer were used for taking measurements at baseline and again at 30 days. The blood samples were collected by trained phlebotomists and analyzed by local pathology laboratories for TC, LDL, HDL, TG and FPG levels. Self-report health conditions were also collected at baseline but not at follow-up. Biometric data were not collected following the 30-day assessment, and so evaluation of the biometric outcomes during the follow-up sessions is beyond the scope of the current study.

### Statistical Analysis

The data were analyzed using IBM™ Statistics (version 19) and expressed as mean ± SD. The extent of the changes (from baseline to post-intervention) in the biometric measures was assessed for non-SDA and SDA separately, using paired *t* tests. Cohen’s d was determined to examine the size of the difference between non-SDA and SDA. One-way between-groups analysis of covariance was also conducted to compare the effectiveness of the intervention between SDA and non-SDA, with the relevant baseline biometric, smoking status and the usual demographic variables age, gender and marital status used as covariates. About 7 % of non-SDA and SDA (*N* = 376 and 114, respectively) did not have follow-up data for some or all biometrics at 30 days, hence the variation in the number (*n*) listed in Table 1 between the biometrics. The McNemar Chi-square test was used to determine the changes in the distribution of participants by SDA status, across the various risk factor categories. Participants’ weight was characterized into risk categories using standard BMI cut points for “normal,” “overweight” and “obese” (NHLBI 2013), BP was classified using the 5th Joint National Committee for Hypertension guidelines (JNC 1993), and FPG was characterized according to conventional “normal,” “impaired” and “diabetic” levels (NCEP 2002). The National Cholesterol Education Program Adult Treatment Panel III (ATP III) classification system (NCEP 2002) was used to categorize the participants for all risk factors, except total cholesterol, for which the Framingham risk classification (Wilson et al. 1998) was used. The Framingham classification includes five cholesterol categories compared to only three in the ATP III classification system and thus allowed a more detailed analysis of the effect of the intervention on the highest risk participants. Metabolic syndrome at baseline and after intervention was classified according to the “harmonized definition” (Alberti et al. 2009). Participants were deemed as having this syndrome if they met three or more of the defining criteria (Alberti et al. 2009). A *P* value of less than 0.05 was considered significant. Confidence intervals (95 %) are also presented. In order to reduce the type 1 error that can occur when simultaneous tests are performed in a data set which is split into risk categories, a Bonferroni correction was applied to each biometric separately. As there were a different number of risk category comparisons for each biometric, the correction applied was 0.05/*n*, where *n* was the number of categories within each biometric. Participants that did not have baseline biometrics were removed.

## Results

Of the 7172 participants, 21.3 % (*n* = 1523) were SDA and 78.7 % (*n* = 5643) were non-SDA. There was no difference between SDA and non-SDA in the distribution of men and women, age or the proportion reported being married. More non-SDA than SDA reported smoking at baseline (Table 1). In terms of baseline biometrics, both non-SDA and SDA were representative of an at-risk population with a mean BMI in the “obese” category and elevated SBP and LDL. Even so, non-SDA had higher mean baseline weight, BMI, TC, LDL, HDL, FPG and pulse rate (Table 1). However, the effect size for these differences was very small to small (Cohen’s *d* range, 0.06–0.19). There were no differences between SDA and non-SDA in mean baseline DBP, TG and TC:HDL ratio.

Non-SDA achieved greater reductions in weight, BMI, TC, LDL and HDL following the intervention, but SDA achieved greater changes in SBP and pulse rate (Table 2). Again, the effect sizes for these differences were only small (Cohen’s *d* range 0.01–0.16). However, there were no differences in changes in DBP, TG, TC:HDL ratio and FPG between non-SDA and SDA (Table 2). The proportion of non-SDA and SDA classified with the metabolic syndrome at baseline decreased significantly at 30 days (non-SDA 44.4–38.1 %, *Χ*^2^(1) = 1973.0, *p* < 0.001; SDA 40.0–31.7 %, *Χ*^2^(1) = 518.4, *p* < 0.001).

Stratification of risk factors showed substantive changes in the distribution of non-SDA and SDA across the various categories, with the largest reductions among participants with the highest risk classifications at baseline (Table 3). Furthermore, while statistical analysis could not be performed for the proportional reduction in non-SDA compared to SDA in each risk category examined separately, more non-SDA than SDA presenting with the highest category for SBP (>160 mmHg), DBP (>100 mmHg), TG (>500 mg/dL) and diabetes (>500 mg/dL) reduced their risk characterization at 30 days (66 vs. 58 %, 77 vs. 52 %, 75 vs. 70 %, 42 vs. 34 %, respectively) (Table 3). Conversely, in the highest risk category for LDL (≥190 mg/dL), more SDA than non-SDA (82 vs. 67 %, respectively) were no longer in this risk category (Table 3).

**Table 3:** Changes in risk factor levels within 30 days by gender and initial risk factor classification, North America, 2006–2012

Risk factor	Non-SDA			SDA		
	Baseline [*N* (%)]	30 days [*N* (%)]^1^	Mean change [(95 % CI) %]^2^	Baseline [*N* (%)]	30 days [*N* (%)]^1^	Mean change [(95 % CI) %]^2^
BMI (kg/m^2^)	*Χ* ^2^ = 561.21, *p* < 0.001			*Χ* ^2^ = 148.05, *p* < 0.001		
<18.5	22 (0.4)	32 (0.6)	−0.14 (−0.40, 0.12)(0.8)	15 (1.1)	14 (1.0)	−0.18 (−0.39, 0.04)(1.0)
18.5–24.9	911 (17.8)	1154 (22.5)	−0.51 (−0.58, −0.45)**(−2.2)	326 (23.9)	397 (29.1)	−0.40 (−0.45, −0.34)**(−1.8)
25–29.9	1534 (29.9)	1612 (31.4)	−0.89 (−0.92, −0.86)**(−3.2)	420 (30.8)	430 (31.5)	−0.80 (−0.86, −0.74)**(−2.9)^†^
≥30	2660 (51.9)	2329 (45.4)	−1.31 (−1.35, −1.27)**(−3.6)	602 (44.2)	522 (38.3)	−1.21 (−1.31, −1.12)**(−3.3)
SBP (mmHg)	*Χ* ^2^ = 763.69, *p* < 0.001			*Χ* ^2^ = 234.47, *p* < 0.001		
<120	1081 (21.2)	1717 (33.7)	2.28 (1.59, 2.97)** (2.0)	327 (24.0)	519 (38.0)	1.54 (0.29, 2.78)* (−1.4)
120–139	2213 (43.5)	2317 (45.5)	−4.56 (−5.07, −4.06)**(−3.5)	598 (43.8)	607 (44.5)	−6.06 (−6.99, −5.12)**(−4.7)^†^
140–160	1359 (26.7)	904 (17.8)	−13.45 (−14.19, −12.71)**(−9.1)	342 (25.1)	198 (14.5)	−13.88 (−15.31, −12.44)**(−9.4)
>160	435 (8.5)	150 (2.9)	−25.54 (−27.37, −23.71)**(−14.8)	97 (7.1)	40 (2.9)	−23.97 (−28.13, −19.81)**(−13.76)
DBP (mmHg)	*Χ* ^2^ = 667.59, *p* < 0.001			*Χ* ^2^ = 157.44, *p* < 0.001		
<80	2333 (45.9)	3184 (62.6)	0.52 (0.15, 0.90)* (−0.7)	674 (49.3)	877 (64.2)	0.02 (−0.65, 0.69) (−0.0)
80–89	1738 (34.2)	1471 (28.9)	−6.02 (−6.41, −5.63)**(−7.2)	451 (33.0)	380 (27.8)	−5.85 (−6.52, −5.18) **(−7.0)
90–100	882 (17.3)	398 (7.8)	−10.82 (−11.38, −10.26)**(−11.5)	208 (15.2)	93 (6.8)	−10.23 (−11.44, −9.02)**(−10.9)
>100	131 (2.6)	31 (0.6)	−21.84 (−25.21, −18.47)**(−19.9)	33 (2.4)	16 (1.2)	−19.73 (−26.75, −12.71)**(−17.6)
TC (mg/dL)	*Χ* ^2^ = 1695.09, *p* < 0.001			*Χ* ^2^ = 372.62, *p* < 0.001		
<160	1101 (21.1)	2071 (39.7)	−7.28 (−8.50, −6.06)**(−5.2)	320 (22.9)	533 (38.1)	−4.37 (−6.46, −2.29)**(−3.1)
160–199	1970 (37.8)	2025 (38.8)	−17.28 (−18.23, −16.33)**(−9.6)	555 (39.6)	550 (39.3)	−13.51 (−15.22, −11.80)**(−7.5)^††^
200–239	1476 (28.3)	879 (16.8)	−27.47 (−28.73, −26.21)**(−12.7)	350 (25.0)	263 (18.8)	−25.95 (−28.24, −23.67)**(−11.9)
240–280	534 (10.2)	207 (4.0)	−40.27 (−42.88, −37.65)**(−15.8)	143 (10.2)	48 (3.4)	−37.95 (−42.52, −33.37)**(−14.9)
>280	137 (2.6)	36 (0.7)	−62.13 (−70.63, −53.63)**(−20.0)	32 (2.3)	6 (0.4)	−54.48 (−70.18, −38.77)**(−18.1)
LDL (mg/dL)	*Χ* ^2^ = 1227.51, *p* < 0.001			*Χ* ^2^ = 254.93, *p* < 0.001		
<100	1687 (33.1)	2525 (49.6)	−5.45 (−6.33, −4.57)**(−6.8)	517 (37.5)	698 (50.7)	−2.51 (−3.97, −1.05)*(−3.1)^†^
100–129	1695 (33.3)	1667 (32.8)	−13.35 (−14.29, −12.42)**(−11.7)	440 (31.9)	421 (30.6)	−11.75 (−13.38, −10.11)**(−10.3)
130–159	1133 (22.3)	669 (13.1)	−22.80 (−24.08, −21.51)**(−16.0)	269 (19.5)	212 (15.4)	−20.84 (−23.08, −18.60)**(−14.5)
160–189	422 (8.3)	178 (3.5)	−30.63 (−32.93, −28.34)**(−17.9)	108 (7.8)	39 (2.8)	−33.75 (−38.40, −29.09)**(−19.7)
≥190	153 (3.0)	51 (1.0)	−46.39 (−51.67, −41.10)**(−22.0)	44 (3.2)	8 (0.6)	−46.09 (−54.48, −37.70)**(−22.3)
HDL (mg/dL)	*Χ* ^2^ = 790.25, *p* < 0.001			*Χ* ^2^ = 112.15, *p* < 0.001		
<40 men, <50 women	2454 (47.1)	3213 (61.6)	−1.80 (−2.03, −1.58)**(−4.7)	685 (48.9)	846 (60.4)	−1.39 (−1.78, 1.00)**(−3.6)
40 men/50 women–59	1626 (31.2)	1314 (25.2)	−4.64 (−4.97, −4.31)**(−9.2)	476 (34.0)	374 (26.7)	−3.43 (−4.01, −2.85)**(−6.7)^†^
≥60	1132 (21.7)	685 (13.1)	−9.63 (−10.22, −9.05)**(−13.5)	239 (17.1)	180 (12.9)	−8.28 (−9.67, −6.90)**(−11.8)
TG (mg/dL)	*Χ* ^2^ = 105.34, *p* < 0.001			*Χ* ^2^ = 49.37, *p* < 0.001		
<150	3368 (64.7)	3587 (68.9)	5.42 (4.24, 6.59)**(−5.7)	913 (65.3)	993 (71.0)	2.63 (0.59, 4.67)* (2.8)
150–199	884 (17.0)	856 (16.4)	−13.20 (−16.59, −9.82)**(−7.7)	227 (16.2)	214 (15.3)	−17.54 (−24.17, −10.91)**(−10.3)
200–499	910 (17.5)	751 (14.4)	−49.37 (−54.32, −44.42)**(−18.4)	249 (17.8)	189 (13.5)	−57.48 (−69.47, −47.48)**(−20.8)
≥500	44 (0.8)	12 (0.2)	−304.77 (−363.13, −246.42)**(47.5)	10 (0.7)	3 (0.2)	−219.91 (−332.72, −107.11)*(−34.2)
FPG (mg/dL)	*Χ* ^2^ = 445.50, *p* < 0.001			Χ^2^ = 67.56, *p* < 0.001		
<100	3230 (62.3)	3769 (72.7)	−1.02 (−1.33, −0.71)** (−1.2)	974 (70.5)	1075 (77.8)	−0.27 (−0.94, 0.39) (−0.3)
100–125	1313 (25.3)	1038 (20.0)	−7.91 (−8.59, −7.24)**(−7.3)	274 (19.8)	217 (15.7)	−6.80 (−7.90, −5.71)**(−6.3)
>125	638 (12.3)	374 (7.2)	−33.06 (−35.89, −30.22)**(−19.9)	133 (9.6)	89 (6.4)	−25.34 (−31.26, −19.41)**(−15.8)

** *p* < 0.001; * Bonferroni correction applied: pre–post-mean change at *p* < 0.0167 level of significance with three risk categories, *p* < 0.0125 with four risk categories, *p* < 0.01 with five risk categories

^1^ *Χ* ^2^ = McNemar Chi-squared statistic: number of participants in that risk category at 30 days; ^††^ difference in change between non-SDA and SDA significant at *p* < 0.001 level of significance; ^†^ Bonferroni correction applied: difference in change between non-SDA and SDA significant at *p* < 0.0167 level of significance with three risk categories, *p* < 0.0125 with four risk categories, *p* < 0.01 with five risk category

^2^% change = [(mean at 30 days − mean at baseline)/mean at baseline] × 100

An analysis of mean changes in the various biometric categories also indicated that non-SDA tended to achieve greater improvements than the SDA. In just 30 days, the majority of non-SDA in the highest risk classifications for DBP, TC, LDL, TG and FPG, but only LDL and TG for SDA were able to show improvement by 20 % or more (Table 3).

## Discussion

Substantial reductions in selected risk factors were achieved in 30 days using the CHIP lifestyle intervention for both SDA and non-SDA, but the reductions were greater for BMI, pulse and blood lipids among non-SDA. These differences go beyond higher baseline levels in non-SDA. Furthermore, in 30 days, the majority of non-SDA in the highest risk classifications for DBP, TC, LDL, TG and FPG, but only LDL and TG for SDA were able to show improvement by 20 % or more. A seemingly adverse outcome of the CHIP intervention is the reduction in HDL among both non-SDA and SDA, which has also been observed in other lifestyle interventions that promote a plant-based eating pattern (Ornish et al. 1998). However, this reduction in HDL is not considered detrimental to the risk of chronic disease when a whole-food plant-based diet is adopted, as discussed by Kent et al. (2013b).

National estimates of mean body weight and BMI for the USA, 1999–2002, are lower than that of the sample of non-SDA in this study (Ogden et al. 2004). Indeed, 82 % of the non-SDA in this study was classified as overweight or obese, while 64 % of the US population was classified as such in 1999–2000 and 69 % in 2011–2012 (CDC and NHANES 2003; CDC 2013). Non-SDA participants in this study faired more positively in terms of the proportion with cholesterol levels >200 mg/dl and LDL levels >130 mg/dl compared to the general US population (Go et al. 2014). However, a greater proportion of non-SDA participants in this study were characterized with diabetes than the 2012 general US population (CDC 2014b).

Compared to SDA in the Adventist Health Study-2, the SDA in the present study were slightly younger (60.3 vs. 57.7 years) and had higher BMI (28.2 vs. 30.1 kg/m^2^), total cholesterol (188.3 vs. 190.2 mg/dl), triglycerides (123.5 vs. 143.3 mg/dl) and FPG (95.6 vs. 97.8 mg/dl) (Professor Gary Fraser, Chief Investigator, AHS-2, personal communication). As a group, SDA have traditionally enjoyed lower rates of many diseases, including obesity, CVD and diabetes than the general population because of their prescriptive lifestyle. This may explain their lower baseline biometrics compared to non-SDA as they were likely following elements of the CHIP intervention before the program commenced. Given this, it is surprising that the differences between SDA and non-SDA at baseline were not greater. However, it should be noted that the CHIP program targets individuals with established risk factors for chronic disease, so those attending would not be representative of the wider church.

Notwithstanding the greater reductions among non-SDA, SDA did achieve substantial risk reductions for most biometrics. More than 50 % of non-SDA and SDA with higher baseline risk levels for SBP, TC, LDL and TG reduced their risk characterization after the 30-day intervention. The changes in TC and LDL levels compare favorably to those achieved by pharmaceutical interventions involving statins (Gould et al. 2007), but without the risk, and are much greater than that expected from dietary interventions aimed at lowering blood lipids (Tang et al. 1998). Furthermore, almost half of non-SDA and one-third of SDA characterized with diabetes reduced this characterization. Of note, risk factor characterization of all biometrics was reduced to at least the next lower level for both non-SDA and SDA, but more so for non-SDA, with some participants reducing two or three levels, particularly if they were in the higher risk categories at baseline. As a result, the amount of change between non-SDA and SDA in the highest baseline levels for BMI, DBP, TC, TG and FPG may not have reached significance because of the small numbers remaining in these risk categories at 30 days.

The relationship between religiosity/spirituality (RS) and health outcomes is still being explored in the literature. Chida et al. (2009) found a lack of association between RS and mortality in people with severe illness, suggesting that the individual’s RS or decreasing RS activity over the years may dilute the effect of RS on health. This may explain why SDA in this study entered the CHIP program with higher biometric risk factors compared to AHS participants who are representative of the US SDA population as a whole. A recent Australian study found that while SDA consumed more health-promoting foods and less processed foods than non-SDA, the gap between SDA and non-SDA food consumption patterns decreased between 1976 and 2005, possibly due to the ‘secularization’ of the participants (Kent and Worsley 2008).

Interestingly, the protective effect on mortality of Christian church activity or attendance has been found to be quite similar to that of organizational activity in general, suggesting that this effect may not be restricted to Christian faiths alone (Chida et al. 2009). The content of the CHIP program does not include any RS material nor SDA propaganda. Rather up to date, scientific information on the prevention and treatment of chronic disease is provided in a socially inclusive and supportive environment. In light of the supportive SDA church environment, Fraser (2003) reported that “when the group culture supports a belief in clear benefits, defines standards of behavior, and then provides skills and opportunities to improve self-efficacy, success becomes more likely. The perception that one’s performance is being observed and compared with community values may be motivating. The teaching of the necessary skills becomes easier when one belongs to a supportive and focused society.” This observation may apply to not only supporters and regular attendees of the SDA church but also individuals who engage in programs within the supportive church environment. The ability of the SDA community to not restrict health and community programs to its own members, and even more so, that non-SDA would accept an invitation to a faith-based program, is noteworthy. Despite the strong positive outcomes achieved by following the CHIP intervention, a strength of this program is that all members of the wider community can meet together and gain the social support needed to experience the benefits.

## Limitations

As the participants were self-selected, they likely entered the program with an elevated readiness for change and hence willingness to engage in the intervention (Norcross et al. 2011). The generalizability of the findings to less motivated populations needs to be determined. A further limitation was the short follow-up time after which the benefits gained by both groups may have been lost. A small New Zealand study found that 106 CHIP participants who returned for follow-up assessment, on average 4 years after completion of the intervention, were able to maintain improvements in most of their biometrics (Kent et al. 2013a). Furthermore, 71 % of participants reported that they were still compliant to the CHIP principles after this time, but it was not clear how this differed for non-SDA and SDA in this preliminary study of only 1 month. As duration of the intervention effects and their costs–benefits, especially in comparison with non-intervention groups, is of key interest to health promoters, the CHIP intervention should be extended, for periods such as 6 months to 5 years.

Information on diet and physical activity was not collected in this study. Similarly, program compliance data were not collected, and, while the biometric changes suggest participants changed their health behaviors, the extent to which this occurred is unknown. Presumably, the participants entered the program with different health behaviors and made varying degrees of change throughout the intervention. A further limitation of this study was that SDA beliefs and attitudes toward the church’s health philosophy, to gauge ‘immunity’ to the secular effects of changes to societal norms and attitudes to health-promoting behaviors, were not collected. Nor was information about non-SDA’s religious beliefs and attitudes toward health in general in this data set. The non-SDA in this data set may have had similar attitudes toward health as SDA, hence their readiness to change their behaviors when provided with the opportunity, knowledge, support and skills to do so. In addition, measures of social support and their relative importance to SDA and non-SDA were not collected in this study. Future studies should gather valid measures of psychosocial factors, including RS, as well as various lifestyle changes made by participants during the CHIP program to elucidate their contribution to the results achieved.

Another limitation is that medication changes and how this differed between SDA and non-SDA were not recorded. Several anecdotal reports from participants in this study indicated that personal physicians decreased doses or even discontinued participants’ medications (e.g., for hypertension, hypercholesterolemia or hyperglycemia) during the 30 days of the intervention. While this is a desirable outcome, reduced medication usage may have caused the results to be understated and to vary between SDA and non-SDA. Further studies are in process to explore the influence of these factors on the outcomes achieved in the program. Despite the limitations in the research design, the current study results are noteworthy, given the size of the sample and the effects observed.

## Conclusion

The CHIP program effectively reduced chronic disease risk factors among both SDA and non-SDA, but more so among non-SDA for many biometrics, with the largest reductions occurring in individuals at greatest risk. This indicates that SDA do not have a monopoly on good health and that positive health outcomes can be achieved by members of the wider community attending this faith-based intervention.
